# First evidence of the deletion in the *pfhrp2* and *pfhrp3* genes in *Plasmodium falciparum* from Equatorial Guinea

**DOI:** 10.1186/s12936-020-03178-9

**Published:** 2020-03-02

**Authors:** Pedro Berzosa, Vicenta González, Laura Taravillo, Alfredo Mayor, María Romay-Barja, Luz García, Policarpo Ncogo, Matilde Riloha, Agustín Benito

**Affiliations:** 1 Malaria Laboratory, National Centre of Tropical Medicine, Institute of Health Carlos III, Madrid, Spain; 2 ISGlobal, Barcelona Ctr. Int. Health Res. (CRESIB), Hospital Clinic-Universitat de Barcelona, Barcelona, Spain; 3 Global Health Programme, Malabo, Equatorial Guinea; 4 Malaria Programme, Ministry of Health and Social Welfare of Equatorial Guinea, Malabo, Equatorial Guinea

**Keywords:** Malaria, Equatorial Guinea, RDTs, *Pfhrp2*/*Pfhrp3*, Deletion

## Abstract

**Background:**

The World Health Organization (WHO) recommends rapid diagnostic tests (RDTs) as a good alternative malaria-diagnosis method in remote parts of sub-Saharan Africa. The majority of commercial RDTs currently available detect the *Plasmodium falciparum* protein histidine-rich protein 2 (PfHRP2). There have also been recent reports of *pfhrp2* gene deletions being found in parasites collected from several African countries. The WHO has concluded that lacking the *pfhrp2* gene must be monitored in Africa. The purpose of the study was to analyse why the samples that were positive by PCR were negative by RDTs and, therefore, to determine whether there have been deletions in the *pfhrp2* and/or *pfhrp3* genes.

**Methods:**

Malaria NM-PCR was carried out on all the samples collected in the field. A group of 128 samples was positive by PCR but negative by RDT; these samples were classified as RDT false-negatives. PCR was carried out for exon2 of *pfhrp2* and *pfhrp3* genes to detect the presence or absence of these two genes. Frequencies with 95% confidence intervals (CIs) were used for prevalence estimates. Associations were assessed by the Chi square test or Fisher´s exact test. The level of significance was set at p ≤ 0.05. Statistical analyses were performed using the software package SPSSv.15.0.

**Results:**

After PCR, 81 samples were identified (4.7%, 95% CI 3.8–5.8) which had deletion in both genes, *pfhrp2* and *pfhrp3*. Overall, however, 11 samples (0.6%, 95% CI 0.36–1.14) had deletion only in *pfhrp2* but not in *pfhrp3*, and 15 (0.9%, 95% CI 0.6–1.5) presented with deletion only in *pfhrp3* but not in *pfhrp2*. Considering the *pfhrp2* gene separately, within the total of 1724 samples, 92 (5.3%, 95% CI 4.37–6.5) had evidence of deletion.

**Conclusion:**

The present study provides the first evidence of deletion in the *pfhrp2* and *pfhrp3* genes in *P. falciparum* isolates from Equatorial Guinea. However, larger studies across different regions within the country and across different seasonal profiles are needed to determine the full extent of *pfhrp2* and *pfhrp3* deletion. It is strongly recommended to implement an active surveillance programme in order to detect any increases in *pfhrp2* and *pfhrp3* deletion frequencies.

## Background

Equatorial Guinea (EG) in Central West Africa is divided into two regions, the Insular Region (Bioko, Annobon) and the Continental Region (Rio Muni). Malaria remains a major public health problem in the country, and EG is a holo-endemic area with year-round transmission [1]. According to official data from EG’s National Malarial Control Programme, the prevalence of *falciparum* malaria in the country (for children between 2 and 14 years old) was 12.5% in 2018. Malaria prevalence on Bioko Island was 10.3 and 46.5% in the Continental Region. The 2018 Malaria Report does not report prevalence for the different species, however, 2011 data for the Continental Region show that 95.2% of malaria infections were *Plasmodium falciparum* and 9.5% *Plasmodium vivax,* with eight cases of mixed infection [2].

The main malaria-control strategy is quick and accurate diagnosis followed by effective treatment [3]. Early and accurate diagnoses are essential for both effective disease management and for proper malaria surveillance. The quality of malaria diagnosis is important in all settings, as misdiagnosis can result in significant morbidity and mortality. Since 2010, the World Health Organization (WHO) has recommended that all patients with suspected malaria should have their diagnoses confirmed by microscopy or a rapid diagnostic test (RDT) before treatment [4]. Microscopy and RDTs are the primary choices for malaria diagnosis in the field. Furthermore, in remote parts of sub-Saharan Africa, RDTs have become the primary tool for the parasitological diagnosis or malaria confirmation [5]. In the absence of well-trained technicians for microscopic diagnosis of malaria in many areas, the WHO recommends RDTs as a good alternative malaria-diagnosis method [6, 7].

RDTs are commonly used in malaria case-management and elimination programmes especially in remotes areas where microscopy facilities are not available [8]. As the tests are easy to perform and provide rapid results (15–20 min), they are exceedingly useful for rapid and malaria diagnosis in most malaria-endemic areas [9]. The majority of commercial RDTs currently available detect the *P. falciparum* protein histidine-rich protein 2 (PfHRP2), which *P. falciparum* only expresses in blood during the ring stage [10]. However, the failure to detect and treat false-negative infection increases the risk that people in a given community can contribute to onward infection through mosquitoes. False negative: that sample which is negative by RDT and positive by another diagnostic method, in this case NM-PCR for malaria.

The antibodies on the test strip detect the PfHRP2 antigen but may cross-react with proteins expressed by another member of the HRP family, PfHRP3, as there are very similar amino acid sequences [9]. Procurement decision-makers’ general preference for PfHRP2-based RDTs is largely based on the findings of a number of studies which report that these tests are both more sensitive and heat-stable than the RDTs that detect other malaria antigens such as *Plasmodium* lactate dehydrogenase, whether pan-pLDH (all species) or *P. falciparum*-specific (Pf-pLDH) or aldolase-based tests [11]. The major drawbacks for RDTs are false positives, because PfHRP2 persists in the blood for several days after an infection has been cleared [12], and false negatives that can be due to *pfhrp2/pfhrp3* gene deletions, have been observed for HRP2 in African field-isolates [13]. In 2010, Gamboa et al. reported the first confirmed identification of *P. falciparum* parasites with *pfhrp2/pfhrp3* gene deletions; these parasites, which expressed neither PfHRP2 nor PfHRP3, were identified in the Peruvian part of the Amazon River basin [9]. There have also been recent reports of HRP2 deletions being found in parasites collected from several African countries, including the Democratic Republic of the Congo, Ghana, Kenya, and Rwanda, in addition to India [5, 14–17]. Importantly, patients with false negatives may not receive treatment at all or may receive it later. Due to the increase in reports of RDT false-negatives in African countries, the WHO has concluded that malaria parasites lacking the *pfhrp2* gene must be rigorously monitored [8].

RDTs were introduced in EG in 2010, although microscopy is still considered the ‘gold standard’ for malaria diagnosis in the country. In 2017, 60,798 RDTs were distributed in EG to different hospitals and health centres [18].

The objective of the present study was to analyse why the samples were positive by nested multiplex-PCR (NM-PCR) and microscopy was negative using RDTs, and, therefore, to determine whether there have been deletions in the *pfhrp2* and/or *pfhrp3* genes that could lead to false negatives.

## Methods

### Study area

**Fig. 1:**
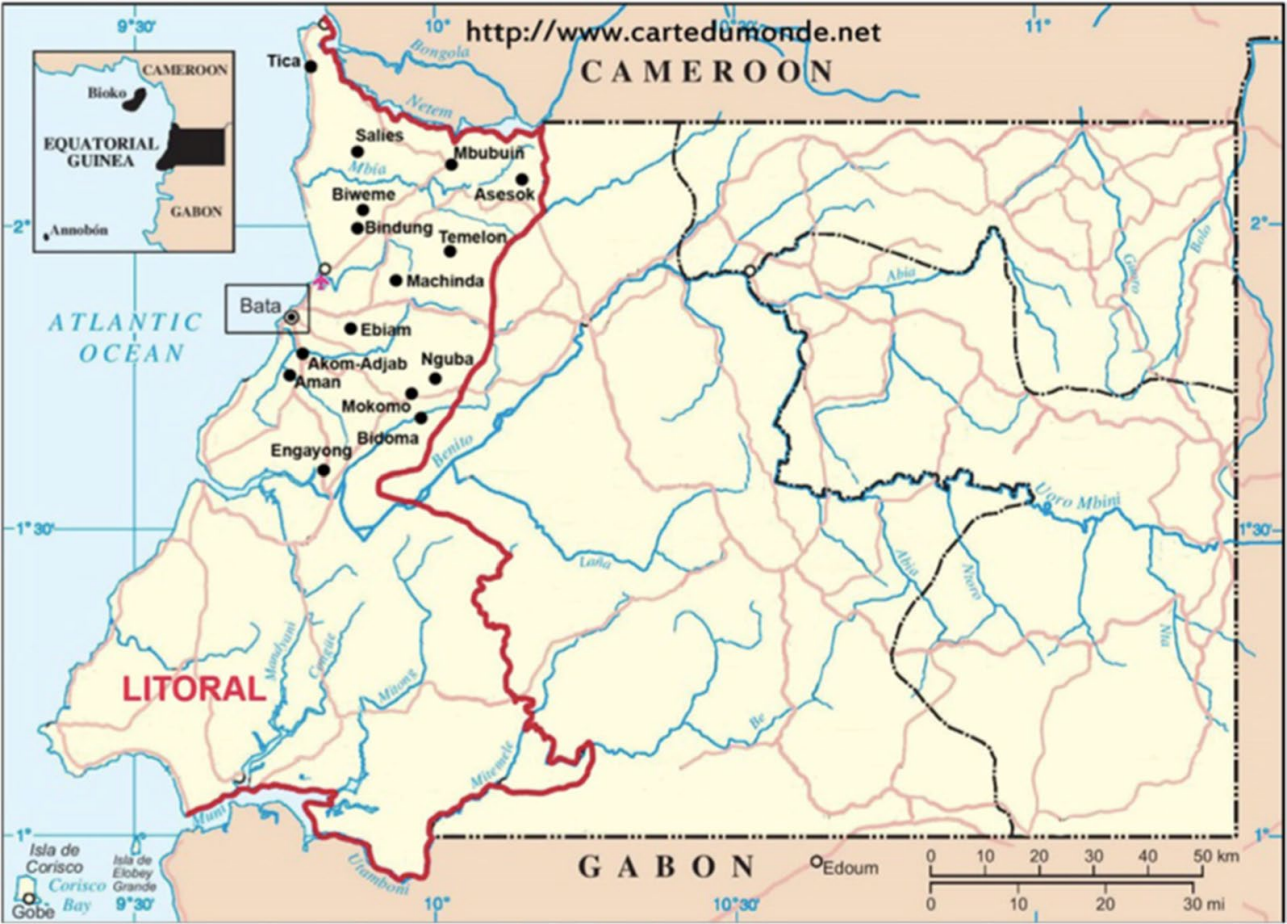
Map of the Continental Region of Equatorial Guinea. The Littoral Province where the sampling took place is highlighted Source https://www.cartedumonde.net, modified. This map was used in Berzosa et al. [20]

The survey was carried out in the district of Bata in the Litoral Province of the Continental Region of EG, located between Cameroon and Gabon (Fig. 1). The region has a tropical climate with two dry seasons (December to March, June to September) alternating with two rainy seasons (March to June, September to December). The mean daily maximum temperatures are 29–32 °C and the minimum temperatures, 19–22 °C.

### Study population

The samples were collected from a cross-sectional survey conducted in June–August 2013 in Bata as part of a project called ‘PREVAMAL’. A total of 1741 individuals (1043 in urban settings and 698 in rural) were recruited [19, 20]. Figure 1 shows the different locations where the samples were collected.

Blood samples were taken from participants’ fingers for malaria diagnosis using both RDTs and microscopy. The blood was spotted on Whatman 903™ paper (GE Healthcare Bio-Sciences Corp.) for further molecular studies. The blood on the filter paper was air dried, stored in double zip-lock plastic bags with silica gel at 4 °C, and subsequently transported to the National Centre for Tropical Medicine, Institute of Health Carlos III, Madrid (Spain) for diagnostic confirmation by PCR.

### Microscopy

The samples were taken in participants’ homes, and the thick and thin slides were also prepared on site. The peripheral blood specimen slides were made immediately after collection on clean, grease-free microscope slides and allowed to air dry. The films were stained with 10% Giemsa solution (Appichem, Panreac ITW Companies) for 10 min, and examined by WHO-certified microscopists from the National Malaria Programme of EG’s Ministry of Health and Social Welfare. After air drying, each slide was subjected to an oil immersion objective lens examination; all fields were examined before declaring a slide negative. For each specimen, the thick films were examined first in order to detect malaria parasites; the thin films of each specimen were only then examined for speciation in those instances when parasites had already been identified in the thick film. The slides were each examined by two microscopists; each specimen was examined independently, and the result was recorded as positive when both microscopists found both evidence of a malaria parasite, and identified the same species. In the event of a discrepancy, a third microscopist also assessed the slide.

### Rapid diagnostic test

The RDT used in situ was the NADAL^®^ Malaria 4 species test (Test cassette) (Nal von Minden, Moers, Germany). This test enables differential diagnosis between *P. falciparum*, *P. vivax*, *Plasmodium malariae* and *Plasmodium ovale* in human whole-blood samples. It detects HRP2-specific proteins for *P. falciparum*, and pLDH-specific proteins for *P. falciparum*, *P. vivax*, *P. malariae*, and *P. ovale*. The test has a sensitivity of 99.7% for *P. falciparum* and 95.5% for non-*P. falciparum* parasites with the microscopic detail of a large droplet, and a specificity of 99.5%. The cut-off level was 1 to 50 parasites/µl of blood for HRP2 and 51 to 100 parasites/µl of blood for pLDH. To perform the malaria test, 5 µl of whole blood is collected with the provided capillary pipette and transferred to the sample well. Four drops of the assay diluent are then added to the diluent well, in accordance with the manufacturer’s protocol. The results are read after 15–20 min; only tests containing the control band are considered valid. Participants whose RDTs produced positive results were immediately offered treatment as set out by the EG national guidelines [21].

### DNA extraction and molecular analysis

The DNA was extracted from the filter paper samples using commercial kits (Speedtools tissue DNA Extraction Kit, Biotools, Spain). A 5-mm diameter punch was used that contained 10 μl of blood.

A. **NM-PCR for the diagnosis of malaria**: The target selected in this NM-PCR is the gene encoding the 18S small sub-unit ribosome RNA (ssrDNA) and includes an internal amplification control to avoid false negatives (18S human rRNA) [22–24]. This was carried out on all the samples collected in the field, including both positives and negatives by microscopy and by RDTs irrespective of the result. After the NM-PCR tests had been completed, a group of 128 samples was identified for further study; these samples were positive using PCR and by microscopy, but negative using RDTs. Therefore, these samples were classified as RDT false-negatives.
B. **Nested PCR for*pfdhfr***, ***pfdhps***, ***pfmdr1*and*pfcrt*genes:** These genes were studied in the RDT false-negative samples, in accordance with the Maryland University protocols [25]. The nested-PCR included the following fragments of each gene: *pfdhfr* (108/164, 51/59), in *pfdhps* (400 and 500), in *pfmdr1* (86/1246) and *pfcrt* (76). This nested-PCR was used as a control for the quality of the DNA. Thus, if all the samples worked in the PCR of these genes, this indicates that the DNA has quality for the PCR. Therefore, if no exon2 amplification fragment is obtained from the *pfhrp2* and *pfhrp3* genes, it was not due to poor DNA quality or other factors, but because there was a true deletion in the *pfhrp2*/*3* genes.
C. **PCR for exon2 of*pfhrp2*and*pfhrp3*genes** The 128 samples with intact parasite-DNA confirmed by NM-PCR and *pfdhfr*, *pfdhps, pfmdr1* and *pfcrt* nested-PCR were used for further amplification of the exon 2 of *pfhrp2* and *pfhrp3* genes. This was to detect the presence or absence of these two genes [5, 6, 26]; this test was performed as described previously, with the same primers but with some minor changes.

**pfhrp2-F1** (5′-CAAAAGGACTTAATTTAAATAAGAG-3′)/**pfhrp2-R1** (5′-AATAAATTTAATGGCGTAGGCA-3′) were designed to anneal to the 5′ and 3′ends of exon 2 of *pfhrp2*. Seminested amplification was performed by use of the primers **pfhrp2-F2** (5′-ATTATTACACGAAACTCAAGCAC-3′) and **pfhrp2-R1**. The same procedures and conditions were used to amplify the *pfhrp3* gene by use of the primers **pfhrp3-F1** (5′-AATGCAAAAGGACTTAATTC-3′), **pfhrp3-R1** (5′-TGGTGTAAGTGATGCGTAGT-3′), and **pfhrp3-F2** (5′-AAATAAGAGATTATTACACGAAAG-3′). These changes included the use of Biotools Hotsplit DNA polymerase (5U/µl) (Biotools B&M Labs, S.A. Madrid, Spain), and the PCR conditions were, 1st PCR y 2nd PCR: 95C for 15 min, followed 30 cycles by 95C for 1 min, 60C for 1 min, and 72C for 1 min and final extension 72C 10 min, for both genes. *Plasmodium falciparum* 3D7 strain parasite was used as a positive control for *pfhrp2* and Dd2 as a negative. This is because 3D7 is known to have all *pfhrp2* and *pfhrp3* genes, as well as the relevant flanking genes, while Dd2 lacks both *pfhrp2* and its flanking genes. All the positive amplifications of *pfhrp2* and *pfhrp3* genes (exon2) were sequenced from both directions using forward and reverse primers of exon2. PCR products were purified with Ilustra exoprostar 1-step (GE Healthcare Life Sciences) in accordance with the manufacturer’s instructions and were used in a standard dye terminator (Big Dye Terminator v3.1 Cycle Sequencing kit); the DNA was then sequenced using an ABI PRISM 3730 XL Analyser. BLAST (Basic Alignment Search Tool) was used for the sequence analysis, and homology with *pfhrp2* and *pfhrp3* of *P. falciparum* were established using MultAlin [27] and Sequence Manipulation Suite [28].

### Statistical analysis

Frequencies with 95% confidence intervals (CIs) were used for prevalence estimates. Associations were assessed by the Chi square test or Fisher´s exact test. The level of significance was set at P ≤ 0.05. Statistical analyses were performed using the software package SPSSv.15.0.

## Results

**Fig. 2:**
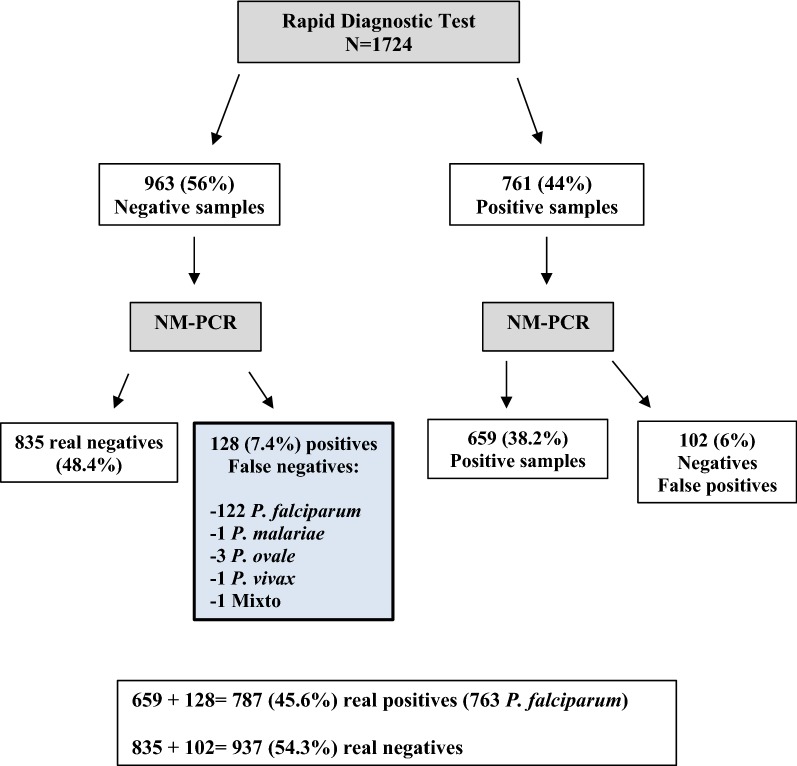
Sample Processing Flowchart. It is observed that 963 samples out 1724 were negatives by RDT and 761 positives. By NM-PCR was detected inside the negative group 128 positive samples (122 Pf, 1 Pm, 3 Po and 1 Pv/Pf), therefore, they were false negatives. The frequency of false negatives by RDT is 7.4%. In these 128 samples were studied the *Pfhrp2* and *Pfhrp3* genes. All these data are published in a previous article Berzosa et al. [20]

A total of 1724 blood samples were diagnosed by microscopy, NM-PCR and RDT [20]. The *Plasmodium* spp. samples marked as negative by both microscopy and RDT were tested by NM-PCR as a quality control of the diagnoses. In this group of negative-by-RDT samples (n = 963), 128 (7.4%) were identified as false negatives by PCR and were diagnosed as: 122 *P. falciparum*, 1 *P. falciparum*/*P. vivax*, 1 *P. malariae*, *1 P. vivax* and 1 *P. ovale*. Figure 2 shows how the 1724 samples were processed. The 128 negative by RDT samples could have been due to a number of possible causes: deletion in the *pfhrp2* or *pfhrp3* genes, technicians’ misinterpretation, or that there is parasitaemia in the sample that is lower than the minimum detection threshold for RDTs.

**Fig. 3:**
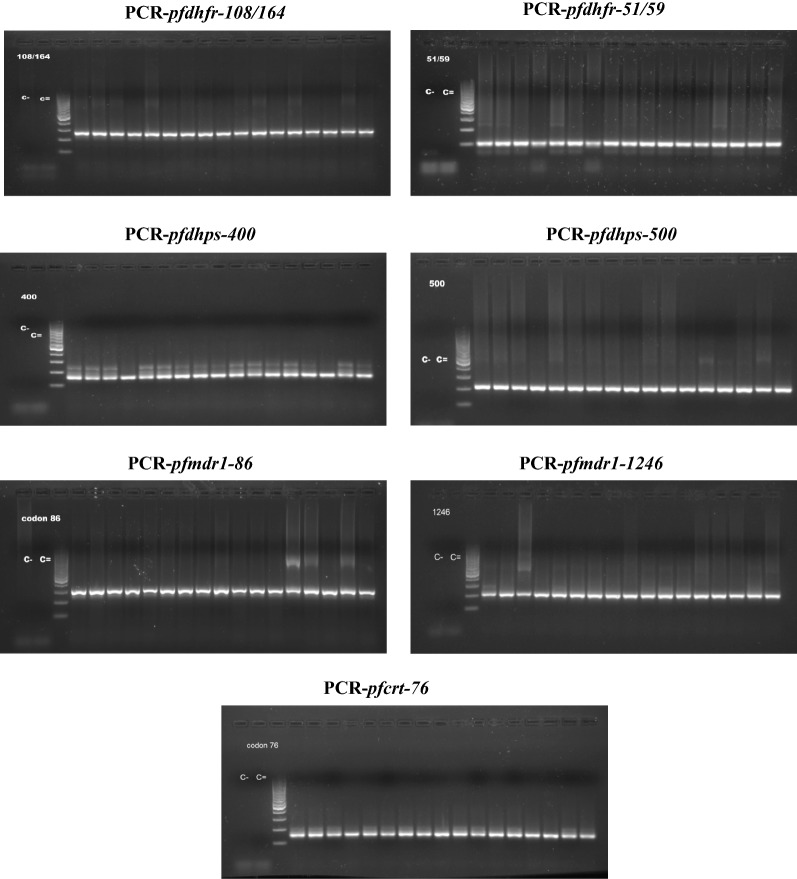
Results of the Nested PCR for *Pfdhfr, Pfdhps, Pfmdr1* and *Pfcrt* genes: amplification appears in all cases, therefore, it indicates that the DNA was well extracted and works correctly in PCR. These PCRs are used as a control, all samples amplified perfectly so when no amplification appears in *pfhrp2*/*3* indicates for sure that there is deletion, it is not a problem with the DNA

**Fig. 4:**
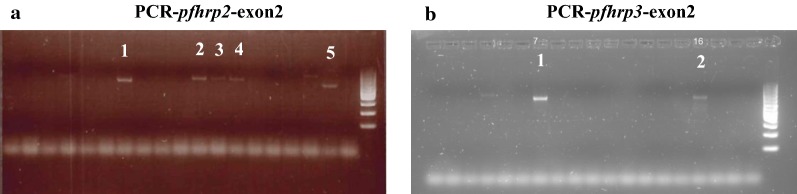
Results of the Nested PCR for *Pfhrp2/3;* the presence of the amplification fragment indicates the presence of the gene: figure (**a**), lines 1/2/3/4/5 (± 814 bp for *Pfhrp2*) and figure (**b**) lines 1/2 (± 719 bp for *Pfhrp3*). When the fragments do not appear indicate that deletion exists; fragments are sequenced to confirm that they correspond to the *pfhrp2/3* genes

The DNA from the 128 false negatives by RDT was amplified correctly by the nested-PCR for the following genes of *P. falciparum* (Fig. 3): *pfdhfr* (108/164 and 51/59, product sizes 254 bp and 113 bp, respectively), *pfdhps* (400 and 500, product sizes 148 bp and 201 bp, respectively), *pfmdr1* (86 and 1246, product sizes 203 bp and 295 bp, respectively) and *pfcrt* (76, product size 145 bp). This indicates that the DNA has been extracted correctly, that it has no inhibition factors for PCR and that it has sufficient concentration to be used successfully in the PCR of exon2 of *pfhdrp2* and *pfhrp3* genes. All the DNA samples were studied for the deletion of *pfhrp2* and *pfhrp3* using PCR (PCR for exon2 of the *pfhrp2* and *pfhrp3* genes); the sizes of the expected fragments, if amplification did occur, were ± 814 bp for *pfhrp2* and ± 719 bp for *pfhrp3*; this determined the presence or absence of these genes in the samples. Figure 4 shows the result of the PCR tests. The decision was made to perform the PCR for exon2 of *pfhrp2*/*3* in all samples that were negative for RDT and positive for NM-PCR, although some samples were diagnosed as non-*falciparum*. In the non-*falciparum* samples, amplification fragment for *pfhrp2*/*3* was not to be obtained. In this way it was also tested the specificity of the PCR for exon2 *pfhrp2*/*3*, as it only amplify these genes of *P. falciparum*. This PCR does not give false positives (amplification fragment with other species of *Plasmodium*), that is, the non-falciparum samples acted as negative controls for the PCR.

After carrying out the PCR on 128 RDT false-negative samples, 5 non-*falciparum* (1 *P. malariae,* 3 *P. ovale,* 1 *P. vivax*) samples were, as expected, negative in the *pfhrp2/pfhrp3*-PCR. The mix sample (*P. falciparum/P. vivax*) was negative in the PCR for these two genes, therefore, was detected deletion in these genes for *P. falciparum*. In the remaining 122 samples which were *P. falciparum*, 81 samples were identified (4.7%, 95% CI 3.8–5.8) out of 1724 which had deletion in both genes (Table 1); therefore, the amplification fragment was absent. Fifteen samples (0.87%, 95% CI 0.53–1.43) had no identifiable deletion in any of the genes studied. In this last case, the expected amplification fragments appeared and were purified and sequenced, and after the comparison in BLAST they were found to have homology with the exon2 of the two genes under study. Overall however, 11 samples (0.6%, 95% CI 0.36–1.14) had deletion only in *pfhrp2* but not in *pfhrp3*, and 15 (0.9%, 95% CI 0.6–1.5) presented with deletion only in *pfhrp3* but not in *pfhrp2*. Considering the *pfhrp2* gene separately (the RDT detects the protein *pfhrp2*), within the total of 1724 samples, 92 (5.3%, 95% CI 4.37–6.5) had evidence of deletion. In the mixed infection (*P. falciparum*/*P. vivax*) according to NM-PCR, neither the *pfhrp2* nor the *pfhrp3* genes were detected.

**Table 1:** Samples in which deletion is detected in *Pfhrp2* and *Pfhrp3* genes

Samples	No of samples	*Pfhrp2*	*Pfhrp3*	N = 1724 (%)	95% CI	N = 763 (%)	95% CI
*P. falciparum* (N = 122)	81	D	D	4.7	3.8–5.8	10.6	8.62–13
	15	ND	ND	0.87	0.53–1.43	1.97	1.19–3.22
	11	D	ND	0.6	0.36–1.14	1.4	0.81–2.56
	15	ND	D	0.87	0.53–1.43	1.9	1.19–3.22
	92	D	NC	5.3	4.37–6.5	12	9.94–14.56
Mixed infection (Pf/Pv) (N = 1)	1	D	D	0.06	1e-04–0.33	0.1	2e-04–0.74

It is observed that in 81 samples appear deletion in both genes; 15 have no deletion in either of them, 11 have deletion only in *Pfhrp2*, 15 only in *Pfhrp3*. Regardless of what happens in *Pfhrp3* (whether there is or not deletion), there are 92 samples with deletion in *Pfhrp2*. In the mixed infection case, is detected deletion in both genes

N = 1724 number of total samples, N = 763 total of *P. falciparum* samples by PCR

*D* deletion/*ND* no deletion/*NC* not considered

If the prevalence of deletion is calculated taking account the number of *P. falciparum* detected by SnM-PCR (763) the frequencies for each case were: deletion in both genes, 10.6% (95% CI 8.62–13); no deletion in any gene, 2% (1.97%; 95% CI 1.19–3.22); deletion in *pfhrp2* but not in *pfhrp3*, 1.4% (95% CI 0.81–2.56); deletion in *pfhrp3* but not in *pfhrp2*, 2% (1.9%; 95% CI 1.19–3.22). Deletion just in *pfhrp2* was 12% (95% CI 9.94–14.56) (Table 1).

## Discussion

This study provides the first evidence of *pfhrp2* and *pfhrp3* deletions in *P. falciparum* in EG. The *pfhrp2* deletion prevalence found in the samples was 5.3%; this prevalence is low when compared to that of Ghana (30%), but is very similar to Mali (5%) [15, 29, 30]. As yet, there are no data available for *pfhrp2* and *pfhrp3* deletion in neighbouring Cameroon and Gabon. The WHO guidelines consider a *pfhrp2* deletion prevalence of 5% as a minimum threshold to change RDT types [31], or, and if not possible, confirm the result of the RDT by another technique, such as microcopy. This study identified 5.3% *pfhrp2* deletion in the regional sample; moreover, this shows that it is now necessary to monitor the deletion of this gene across the whole country in order to obtain a complete picture of the deletions occurring with these genes. It is important to remember that this study was carried out in a district of EG’s continental region; it is evident that the study needs to be extended to cover the country in its entirety.

Deletions in *pfhrp3* were also detected in the study, although the RDT used in EG and for this study was not designed to detect *pfhrp3* proteins. In most settings, genetic mutations like *pfhrp2/pfhrp3* deletion in parasites are unlikely to be the main cause of RDT false-negatives unlike in this study, and more studies are required to establish the true prevalence of these mutations in EG. In fact, there were some samples that were RDT false-negatives which were found to be positive using NM-PCR, but without any detectable deletion in the *phrp2* and *pfhrp3* genes. Therefore, these results might be due to problems with the RDT used itself, or as result of operator error when carrying out tests and/or interpretation RDT results; all of which could result in false-negatives [32].

Attributing false-negatives to *pfhrp2/pfhrp3* deletion has significant implications for public health policy. Once it has been established that the threshold has been passed, alternative RDTs will have to be procured and case-management decisions will have to be revised, with retraining in the use of the new RDTs. Investigation into such deletions must be carried out systematically and accurately [7]. If *pfhrp*2 deletions are found to be prevalent among symptomatic individuals (the lower 95% CI is still above 5%), as is the case, for example, in Eritrea and several countries in South America (Brazil, Colombia, Peru), national malaria control programmes will have to switch to RDTs that do not exclusively rely on PfHRP2 to detect *P. falciparum*. A 5% threshold was selected by WHO because it is somewhere around this point that the proportion of cases missed by PfHRP2 RDTs due to non-*hrp2* expression is likely to be greater than that which would be missed by using less-sensitive pLDH-based RDTs. A recommendation to switch can be further informed by mathematical modelling which shows whether parasites lacking *pfhrp2* genes will spread under PfHRP2-only RDT pressure; policy makers may also decide to switch because of the complexity of procuring multiple RDTs and training staff in their use. In general, any change should be applied nationwide, although roll-out might be prioritized on the basis of PfHRP2-deletion prevalence in a given region [33]. Where microscopy is available, services should be strengthened to ensure that parasitological confirmation continues during the transition to new RDTs, and in order to investigate new, suspected PfHRP2/PfHRP3-deleted parasite foci.

Excessive use of *pfhrp*-based RDTs might enhance the selection of *P. falciparum* isolates with *pfhrp2* deletion, especially in endemic areas where *pfhrp2* deletion is present, as the case in EG. Previous reports have also shown that *pfhrp3* deletion can be an early warning sign for *pfhrp2* deletion. Thus, it is important to monitor the presence of parasites with *pfhrp2* deletions to avoid RDT false-negatives, as well as *pfhrp3* deletions to act as an early warning, which offers public health bodies an opportunity to step up monitoring efforts and consider longer term contingency plans [8, 9].

## Conclusion

The RDTs used in this study detected the majority of *P. falciparum* infections as well as those from other species. Regarding the deletion of the genes, it is strongly recommended to implement an active surveillance programme in order to detect any increases in *pfhrp2* and *pfhrp3* deletion frequencies. Although there are false negatives due to causes other than deletion of these genes, a surveillance programme is critical due to the level of frequencies of deletion detected in the study. Surveillance could be implemented in different regions and different seasonal profiles, to determine the full extent of *pfhrp2* and *pfhrp3* deletion.

To be able to control malaria, it is essential to have good diagnostic tools on the front line. To this end, the present study provides the first evidence of deletion in the *pfhrp2* and *pfhrp3* genes in *P. falciparum* isolates from EG. If frequency of deletion increases over time in the country, it might be important to think about changing the type of RDTs used.

## Data Availability

Not applicable.

## Abbreviations

RDTs: Rapid diagnostic tests
HRP: Histidine rich protein
CI: Confidence interval
EG: Equatorial Guinea
PCR: Polymerase chain reaction
NM-PCR: Nested multiplex PCR
N-PCR: Nested PCR
PfHRP2: *Plasmodium falciparum* histidine rich protein 2
PfHRP3: *Plasmodium falciparum* histidine rich protein 3
Pf-pLDH: *Plasmodium falciparum* lactate dehydrogenase
*pfdhfr*: *Plasmodium falciparum* dihydrofolate reductase gene
*pfdhps*: *Plasmodium falciparum* dihydropteroate synthase gene
*pfmdr1*: *Plasmodium falciparum* multidrug resistant 1 gene
*pfcrt*: *Plasmodium falciparum* chloroquine resistance transporter gene

## References

[1] Benito A, Roche J, Molina R, Amela C, Alvar J (1994). Application and evaluation of QBC malaria diagnosis in a holoendemic area. Appl Parasitol.

[2] Mendes C, Dias F, Figueiredo J, Mora VG, Cano J, de Sousa B (2011). Duffy negative antigen is no longer a barrier to *Plasmodium vivax* molecular evidences from the African West Coast (Angola and Equatorial Guinea). PLoS Negl Trop Dis..

[3] Gerstl S, Dunkley S, Mukhtar A, De Smet M, Baker S, Maikere J (2010). Assessment of two malaria rapid diagnostic tests in children under five years of age, with follow-up of false-positive pLDH test results, in a hyperendemic falciparum malaria area, Sierra Leone. Malar J..

[4] WHO. Guidelines for the treatment of malaria. Geneva: World Health Organization, 2010. http://apps.who.int/iris/bitstream/. 25473692

[5] Kozycki CT, Umulisa N, Rulisa S, Mwikarago EI, Musabyimana JP, Habimana JP (2017). False-negative malaria rapid diagnostic tests in Rwanda: impact of *Plasmodium falciparum* isolates lacking *hrp2* and declining malaria transmission. Malar J..

[6] Ugah UI, Alo MN, Owolabi JO, Okata-Nwali OD, Ekejindu IM, Ibeh N (2017). Evaluation of the utility value of three diagnostic methods in the detection of malaria parasites in endemic area. Malar J..

[7] Kim SH, Nam MH, Roh KH, Park HC, Nam DH (2008). Evaluation of a rapid diagnostic test specific for *Plasmodium vivax*. Trop Med Int Health..

[8] Gupta H, Matambisso G, Galatas B, Cisteró P, Nhamussua L, Simone W (2017). Correction to: Molecular surveillance of *pfhrp2* and *pfhrp3* deletions in *Plasmodium falciparum* isolates from Mozambique. Malar J..

[9] Gamboa D, Ho MF, Bendezu J, Torres K, Chiodoni PL, Barnwell J (2010). A large proportion of *P. falciparum* isolates in the Amazon region of Peru lack *pfhrp2* and *pfhrp3*: implications for malaria rapid diagnostic tests. PLoS One..

[10] Bharti KP, Singh CH, Krishna S, Nema S, Ahmad A, Udhayakumar V (2017). Sequence variation in *Plasmodium falciparum* Histidine Rich Proteins 2 and 3 in Indian isolates: implications for malaria rapid diagnostic test performance. Sci Rep..

[11] Global Malaria Programme (Mayo 2016 revision September 2017).

[12] Humar A, Ohrt C, Harrington MA, Pillai D, Kain KC (1997). Parasight test compared with the polymerase chain reaction and microscopy for the diagnosis of *Plasmodium falciparum* malaria in travelers. Am J Trop Med Hyg.

[13] Berhane A, Mihreteab S, Mohammed S, Embaye G, Hagos F, Zehaie A, et al. PfHRP2 Detecting malaria RDTs: alarming false negative results in Eritrea 2016. American Society of Tropical Medicine and Hygiene 65th Annual Conference. ASTMH 2016;95(Suppl.5). Poster 879.

[14] Parr JB, Verity R, Doctor SM, Janko M, Carey-Ewend K, Turmanet BJ (2017). pfhrp2-deleted *Plasmodium falciparum* parasites in the Democratic Republic of the Congo: a national cross-sectional survey. J Infect Dis.

[15] Amoah LE, Abankwa J, Oppong A (2016). *Plasmodium falciparum* histidine rich protein-2 diversity and the implications for PfHRP 2-based malaria rapid diagnostic tests in Ghana. Malar J..

[16] Beshir KB, Sepulveda N, Bharmal J, Robinson A, Mwanguzi J, Obukosia Busula A (2017). *Plasmodium falciparum* parasites with 1 histidine-rich protein 2 (*pfhrp2*) and *pfhrp*3 gene deletions in two endemic regions of Kenya. Sci Rep..

[17] Bharti PK, Singh CH, Ahmad A, Krishna S, Udhayakumar V, Singh N (2016). Prevalence of *pfhrp2* and/or *pfhrp3* gene deletion in *Plasmodium falciparum* population in eight highly endemic states in India. PLoS ONE.

[18] WHO (2018). World malaria report.

[19] Romay-Barja M, Jarrin I, Ncogo P, Nseng G, Sagrado MJ, Santana-Morales MA (2015). Correction: rural-urban differences in household treatment-seeking behaviour for suspected malaria in children at Bata District, Equatorial Guinea. PLoS ONE..

[20] Berzosa P, de Lucio A, Romay-Barja M, Herrador Z, González V, García L (2018). Comparison of three diagnostic methods (microscopy, RDT, and PCR) for the detection of malaria parasites in representative samples from Equatorial Guinea. Malar J..

[21] Ncogo P, Herrador Z, Romay-Barja M, García-Carrasco E, Nseng G, Berzosa P (2015). Malaria prevalence in Bata district, Equatorial Guinea: a cross–sectional study. Malar J..

[22] Ta TH, Hisam S, Lanza M, Jiram AI, Ismail N, Rubio JM (2014). First case of a naturally acquired human infection with *Plasmodium cynomolgi*. Malar J..

[23] Rubio JM, Post RJ, van Leeuwen WM, Henry MC, Lindergard G, Hommel M (2002). Alternative polymerase chain reaction method to identify *Plasmodium* species in human blood samples: the semi-nested multiplex malaria PCR (SnM-PCR). Trans R Soc Trop Med Hyg.

[24] Ta TH, Ali-Tammam M, Lanza M, Rubio JM, Merino L (2011). Detection and identification of *Plasmodium* species by nested multiplex PCR. Manual of molecular methods for microbiological studies.

[25] http://www.medschool.umaryland.edu/malaria/Protocols/.

[26] Baker J, McCarthy J, Gatton M, Kyle DE, Belizario V, Luchavez J (2005). Genetic diversity of *Plasmodium falciparum* histidine-rich protein 2 (PfHRP2) and its effect on the performance of PfHRP2-based rapid diagnostic tests. J Infect Dis.

[27] Corpet F (1988). Multiple sequence alignment with hierarchical clustering. Nucleic Acids Res.

[28] www.bioinformatics.org/sms2/.

[29] Koita OA, Doumbo OK, Ouattara A, Tall LK, Konaré A, Diakité M (2012). False-negative rapid diagnostic tests for malaria and deletion of the histidine-rich repeat region of the hrp2 gene. Am J Trop Med Hyg.

[30] Owusu EDA, Djonor SK, Brown CA, Grobusch MP, Mens PF (2018). *Plasmodium falciparum* diagnostic tools in HIV-positive under-5-year-olds in two ART clinics in Ghana: are there missed infections?. Malar J..

[31] WHO. *Plasmodium falciparum* hrp2/3gene deletions. Malaria Policy Advisory Committee Meeting. Geneva: World Health Organization; 2016. http://www.who.int/malaria/mpac/mpac-sept2016-hrp2-consultation-short-report.session7.pdf.

[32] Kahama-Maro J, D’Acremont V, Mtasiwa D, Genton B, Lengeler C (2011). Low quality of routine microscopy for malaria at different levels of the health system in Dar es Salaam. Malar J.

[33] Global Malaria Programme. False-negative RDT results and implications of new reports of *P. falciparum histidine*-*rich protein 2/3* gene deletions. May 2016 (Rev Sept 2017). World Health Organization.

